# Durable ventricular assist device as salvage therapy for recurrent free wall rupture: Expanding the indication of the durable ventricular assist device

**DOI:** 10.1016/j.xjtc.2025.10.029

**Published:** 2025-11-21

**Authors:** Min-Yuan Liu, Chih-Hsien Wang, Ling-Yi Wei, Heng-Wen Chou, Hsi-Yu Yu, Yih-Sharng Chen

**Affiliations:** Division of Cardiovascular Surgery, Department of Surgery, National Taiwan University Hospital, College of Medicine, National Taiwan University, Taipei, Taiwan

**Figure:**
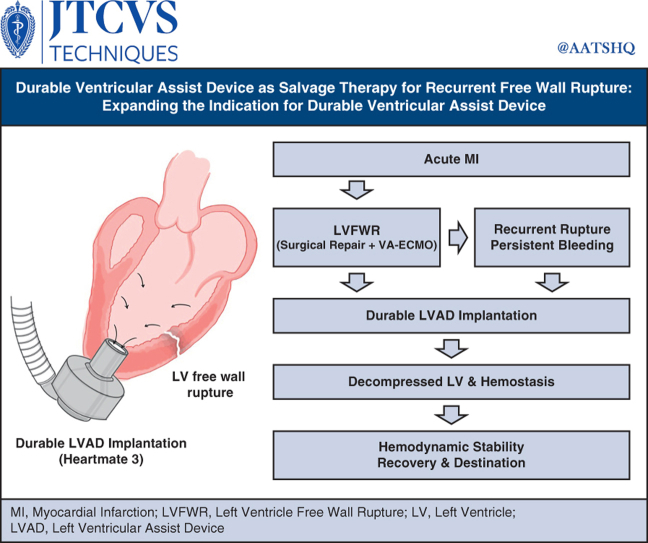

**Figure:**
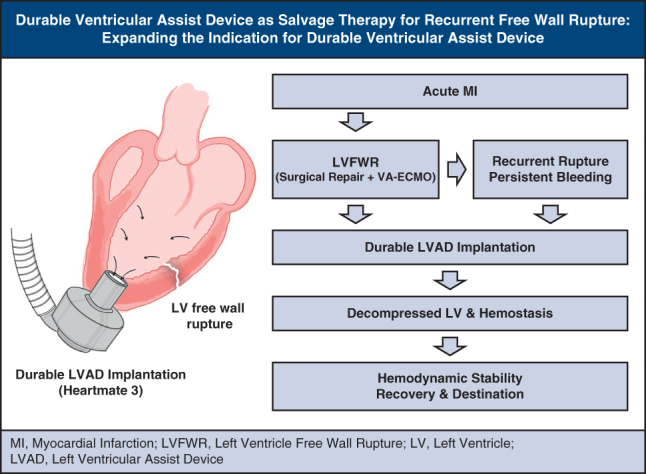
Durable LVAD as salvage therapy for recurrent post-infarction free wall rupture.

**Central Message**

Durable ventricular assist device may represent a viable salvage strategy for recurrent LVFWR, expanding its indication in cases in which conventional interventions fail.

Left ventricular free-wall rupture (LVFWR) is a rare but often fatal complication of acute myocardial infarction (AMI) that typically presents with tamponade and circulatory collapse, requiring immediate surgical control; recurrent rupture is particularly challenging. We describe an inferior ST-segment elevation myocardial infarction case complicated by recurrent LVFWR that was initially repaired and supported with venoarterial (VA) extracorporeal membrane oxygenation (ECMO), but ongoing hemorrhage and myocardial friability necessitated definitive unloading with a HeartMate 3 (HM3) left ventricular assist device (LVAD; Abbott), leading to stabilization and recovery. This experience supports durable mechanical unloading as a salvage strategy when conventional repair is insufficient; in carefully selected high-risk patients, devices such as the Impella 5.5 (Abiomed, Johnson & Johnson MedTech) or HM3 may stabilize hemodynamics and facilitate myocardial recovery.

## Clinical Summary

A 79-year-old man with type 2 diabetes, hypertension, dyslipidemia, and previous percutaneous coronary intervention to the left anterior descending (coronary artery) presented with acute chest tightness and bradycardia. An electrocardiogram showed an inferior ST-segment elevation myocardial infarction with complete atrioventricular block. Echocardiogram demonstrated a left ventricular ejection fraction of 38.6%, 4-chamber dilation (left ventricular end-diastolic diameter/end-systolic diameter 54/44 mm), and inferior-posterior hypokinesis. Emergent percutaneous coronary intervention to the distal right coronary artery was performed with temporary transvenous pacing, and he was admitted to the intensive care unit.

On hospital day 6, he had cardiac arrest with pulseless electrical activity. Extracorporeal cardiopulmonary resuscitation was initiated, although initial VA-ECMO flow was suboptimal. Bedside echocardiography showed a massive pericardial effusion, and emergent subxiphoid drainage promptly restored ECMO flow. Surgical exploration identified a 3-mm posterior LVFWR, which was repaired with a pledgeted 2-0 PROLENE and AQUABRID surgical sealant (Terumo). The sternum was initially left open and closed on hospital day 8.

**Figure 1:**
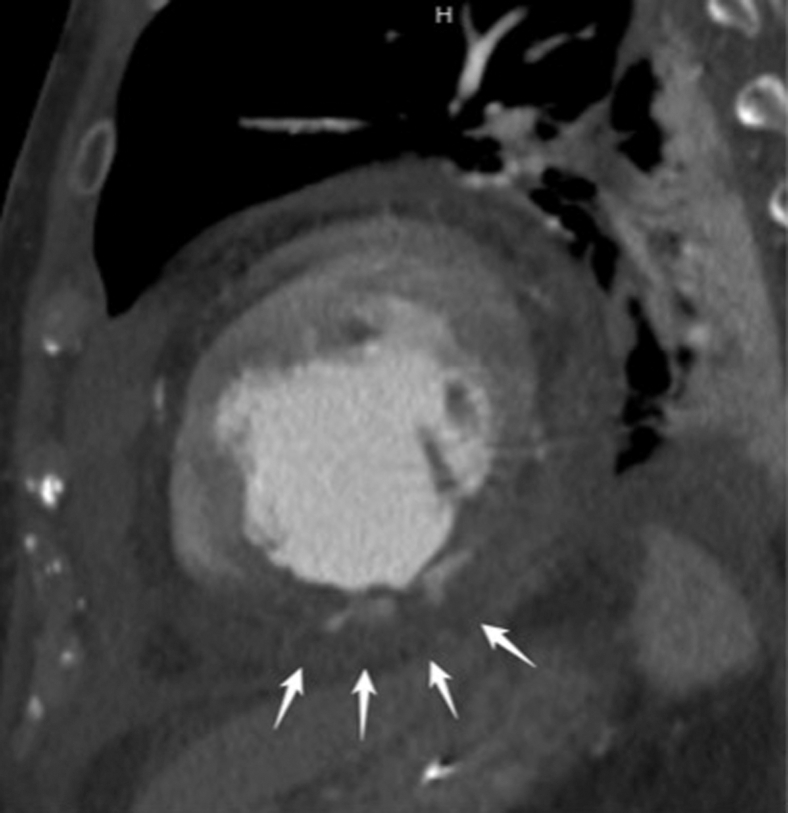
Recurrent hemorrhagic chest-tube output prompted contrast-enhanced computed tomography revealing active contrast extravasation from the posterior LV wall (*white arrows*).

On hospital day 11, recurrent hemorrhagic chest-tube output prompted a computed tomography scan, which revealed active contrast extravasation from the posterior left ventricular (LV) wall (Figure 1). Re-exploration (Video 1) confirmed bleeding at the previous repair. Additional 2-0 pledgeted PROLENE sutures and AQUABRID were applied, but the myocardium was markedly friable—gelatinous, edematous infarcted tissue with poor suture purchase—posing a high re-rupture risk. Given ongoing bleeding and worsening LV function, an HM3 LVAD (Abbott) was implanted; the sewing ring was reinforced with 2-0 PROLENE and felt pledgets. VA-ECMO was weaned intraoperatively; HM3 was set at 4200 rpm with flows 2.0 to 2.5 L/min.

**Figure 2:**
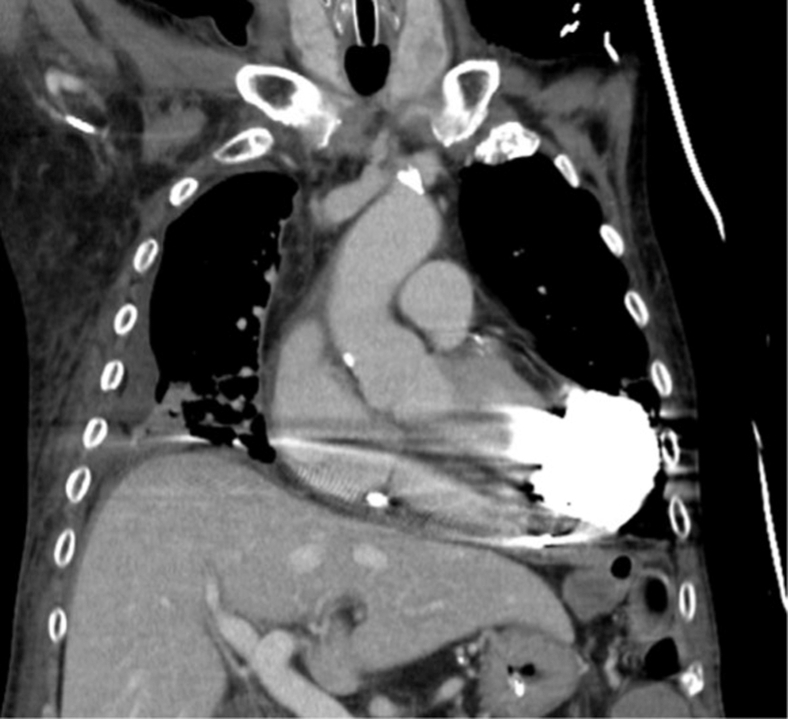
HeartMate 3 was implanted as a salvage strategy for recurrent left ventricular free-wall rupture. Follow-up contrast-enhanced computed tomography confirmed appropriate device positioning for left ventricular decompression.

The patient subsequently stabilized, was extubated on hospital day 14 (post-HM3 day 3), transferred to the general ward on day 17 (post-HM3 day 6), and discharged on day 132 (post-HM3 day 121) after treatment for other medical issues and rehabilitation. Warfarin was initiated on post-HM3 day 3 once chest-tube output was minimal (target international normalized ratio ≈ 2.0); antiplatelet therapy was withheld because of previous gastrointestinal bleeding and concern for recurrent LV hemorrhage, despite the previous right coronary artery stent. At discharge, his blood pressure was 99/86 mm Hg with HM3 settings: speed 5000 rpm, flow 3.0 to 3.3 L/min, pulsatility index 4.5 to 6.7, and power 3.3 W (Figure 2). On outpatient follow-up, future explantation was discussed but deferred by the family; antithrombotic therapy remained warfarin monotherapy (target international normalized ratio ≈ 2.0).

## Discussion

LVFWR is a rare but catastrophic complication of transmural AMI with extensive necrosis. Prognosis is poor without prompt intervention—especially in blow-out rupture—although subacute “oozing” forms may still progress to hemodynamic collapse. Management is primarily surgical (direct suture repair, infarctectomy, or patch reconstruction with synthetic or biologic materials). In friable myocardium, sutureless bioadhesive patches (eg, TachoSil) can be effective. Although conservative management has been described in select cases, it is typically reserved for patients with extreme surgical risk, and the optimal criteria remain undefined.^1^

The advent of mechanical circulatory support (MCS) has markedly expanded therapeutic options for LVFWR. Although VA-ECMO offers partial circulatory support and preserves end-organ perfusion, it may increase left ventricular end-diastolic pressure, thereby exacerbating bleeding risk. In contrast, left ventricular unloading devices such as the Impella 5.5 and LVADs, including HM3, can achieve hemodynamic stabilization, reduce wall stress, and enhance systemic perfusion.^2^^,^^3^ In addition, temporary surgical unloading can also be achieved using a cannula placed through the right superior pulmonary vein into the left atrium or across the mitral valve into the left ventricle, connected to a centrifugal pump with outflow to the ascending aorta (left atrium-aorta or left ventricle-aorta circuit). In our case, National Health Insurance constraints prevented timely access to Impella 5.5, whereas HM3 was immediately available. The patient, aged 79 years, was ineligible for transplant under local criteria. Given persistent hemorrhage and the need for definitive unloading alongside hemostatic control—after shared decision-making with the family—we proceeded with a straight-to-durable LVAD. Impella 5.5 remains a reasonable bridge-to-decision/recovery choice when available.

Emerging data have suggested that early left ventricular unloading in AMI may attenuate infarct size, mitigate reperfusion injury, and reduce the risk of mechanical complications. In cases of LVFWR, MCS may serve as a bridge to recovery or definitive surgical treatment, particularly when conventional repair is insufficient or delayed.^2^, ^3^, ^4^, ^5^ Durable MCS or heart transplantation should be strongly evaluated as part of the therapeutic approach when conventional surgical options are not viable.^2^

## Conflict of Interest Statement

The authors reported no conflicts of interest.

The *Journal* policy requires editors and reviewers to disclose conflicts of interest and to decline handling or reviewing manuscripts for which they may have a conflict of interest. The editors and reviewers of this article have no conflicts of interest.

## Supplementary Data

Video 1 Emergent reoperation confirmed persistent bleeding at the previous repair site. Video available at: https://www.jtcvs.org/article/S2666-2507(25)00540-1/fulltext.
