# Supplementary figures and images for: Durable ventricular assist device as salvage therapy for recurrent free wall rupture: Expanding the indication of the durable ventricular assist device
Source: JTCVS Tech. 2025 Nov 21;35:102159. doi: 10.1016/j.xjtc.2025.10.029 (PMC12881785; doi:10.1016/j.xjtc.2025.10.029)

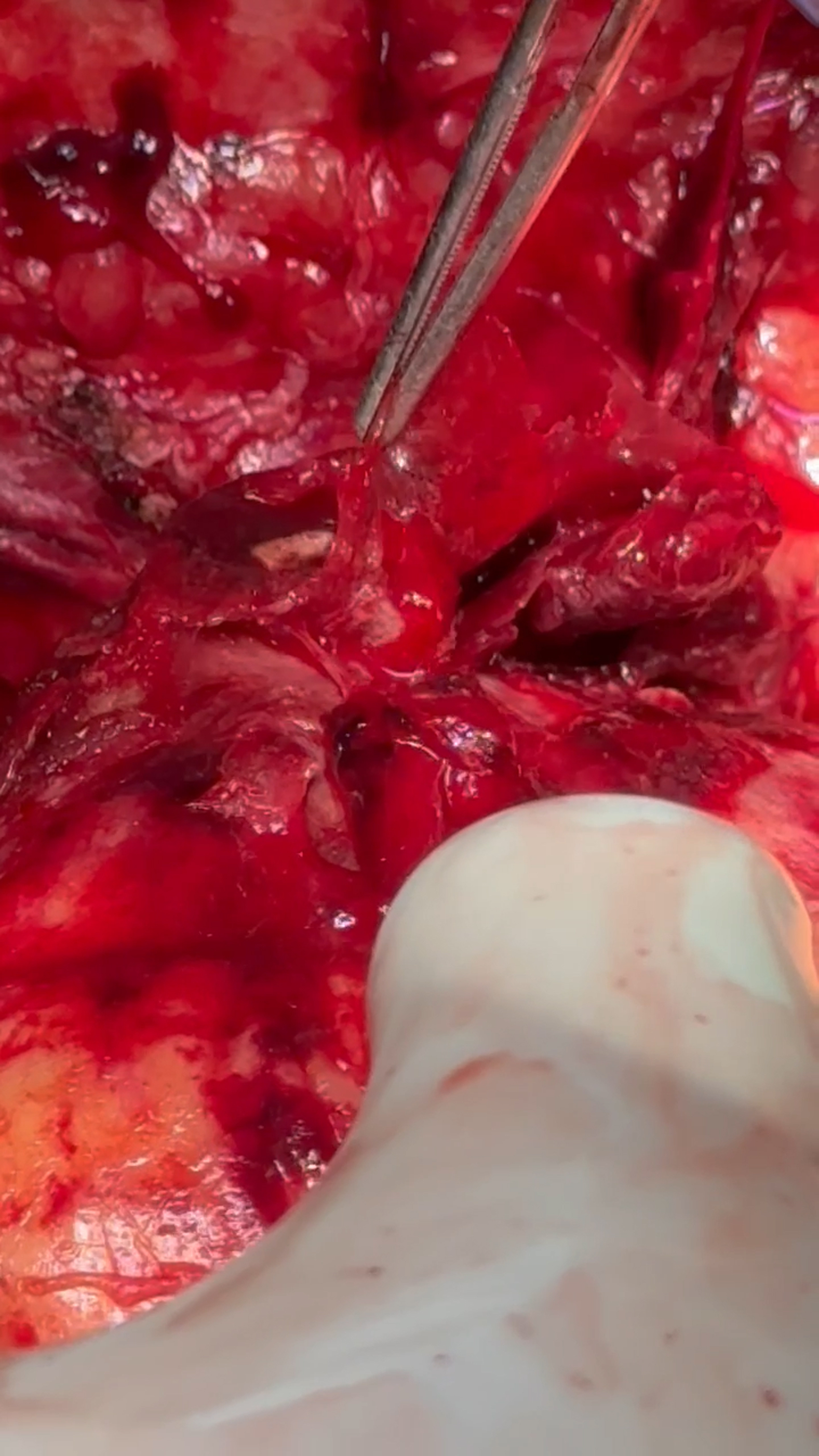

Supplement: Video 1 — Emergent reoperation confirmed persistent bleeding at the previous repair site. Video available at: https://www.jtcvs.org/article/S2666-2507(25)00540-1/fulltext. [file fx2.jpg]
